# Topics of Public Interest

**Published:** 1915-12

**Authors:** 


### TOPICS OF PUBLIC INTEREST.

---

**Abstracts of Official Rulings Under the New Federal Narcotic Law:**

**Administration, External and Internal.** Liniments, ointments, or other preparations containing drugs not specifically exempt, used for oral, nasal, aural, ocular, rectal, urethral, or vaginal administration are not in such cases used externally and are therefore not exempt from the provisions of this law.

**Attendance (personal) Definition of.** A physician, dentist, or veterinarian must actually be absent from his office and in personal attendance upon a patient in order to come within the exemption of section 2, paragraph A, of this law.

**Charity Organizations.** Not supported solely by the state, county or municipality, must register and pay the special tax and keep a record of drugs dispensed or distributed.

**Consumers Obtaining Drugs.** A consumer, as such, will not be permitted to register under this law and can only obtain a supply of such drugs through a duly registered physician, dentist or veterinarian.

**Drugs Dispensed, Record of.** A physician or dentist who administers minute quantities of drugs coming within the scope of this law in his office may keep a record of the date when a stock solution is exhausted without keeping a record of the name and address of each patient to whom such drugs are administered. This plan will be allowed, however, only in cases of those physicians and dentists who use minute quantities of these drugs, such as oculists, aurists and other specialists; but where a physician engaged in a general practice otherwise administers such drugs it will be necessary for him to keep a record of the name and address of the patient, of all drugs dispensed, distributed or administered in his office, and of such drugs left with a patient to be taken in his absence. Only such drugs as are personally administered by a physician to a patient when away from his office are exempt from record.

**Educational Institutions.** Any department of a university, college, or other educational institution using drugs coming within the scope of this law must register with the collector of internal revenue and pay the special tax. The dean of each department should sign the application for registry and the order blanks used to obtain a supply of these drugs. Such drugs used in a dental infirmary or laboratory should be recorded in a book kept for that purpose.

**Exemption From Registration.** Under the act, government, state, county and municipal officers, lawfully engaged in purchasing drugs, etc., specified in the act for the various departments of the army and navy, the public health service, and for government, state, territorial, district, county, municipal or insular hospitals or prisons are held to be exempt under section 1 and paragraph (d) of section 2 from registry and special tax to purchase and use of such drugs and to the keeping of records of the same. Any such officers, however, engaged in private practice must register, pay special tax and keep the records, and comply with all the requirements of the law and regulations.

Hospitals and sanatoriums must keep a record of drugs dispensed, distributed or administered therein.

Inventories must be retained by person making same and not sent to the collector of internal revenue or the Treasury Department. Such inventories must be sworn to.

**Name in Full—Meaning.** A physician may sign prescriptions calling for drugs coming within the scope of this law the same as he would sign a check or legal document, i.e., J. H. Smith, John H. Smith, or John Henry Smith.

**Nurses, Status of.** Not allowed to register and can only have narcotic drugs in their possession under direction of registered physician. Can only obtain supplies of such drugs upon registered physician's prescription and only when nursing patient of such physician.

**Partnerships of Physicians.** Where two or more physicians, dentists or veterinary surgeons are in partnership, doing business under a firm name, it is necessary for the firm to be registered, the firm registry number to be indicated in ordering any of the drugs for use in the office practice of the members of the firm, each individual physician, dentist or veterinary surgeon in such partnership should register and pay the special tax under his own name, if also engaged in private practice.

**Physicians, Dentists and Veterinarians Practicing in More Than One District.** If maintaining an office in more than one internal revenue district must register in each district. If not maintaining more than one office registration in one district permits him to practice in any other district with but one registration.

**Prescription Blanks.** A physician, dentist or veterinary surgeon can make use of any prescription blank, provided the same is properly dated and signed and has indicated thereon the physician's address, his registry number, and the name and address of the person for whom such prescription is written. The government does not furnish a form upon which prescriptions may be written and the special order form can not be used for this purpose.

**Prescriptions, Partial Filling of.** Original prescriptions only can be lawfully filled by druggists, and the partial filling of such prescriptions, from time to time, where large quantities of drugs have been prescribed will, under no circumstances, be permitted.

**Refilling Prescriptions.** Only original prescriptions can be filled by druggists and apothecaries and can not be refilled without violating this law.

**Registration, Who Eligible For.** The following persons legitimately engaged in the practice of their profession and dealers allowed by the state laws to handle narcotic drugs are eligible to registry under this law: Persons engaged in the practice of medicine and surgery, persons engaged in the practice of dentistry, persons engaged in the practice of veterinary medicine and surgery, persons engaged in the importation and sale of drugs, persons engaged in the manufacture and sale of drugs at wholesale, persons engaged in the manufacture and sale of drugs at retail.

An osteopath, therefore, or other person heretofore administering these drugs, if not classed as a physician in the state in which he resides, would not be permitted to register under this law.

**Special Tax Stamps.** Must be posted in a conspicuous place by every person registering under this law.

DAVID A. GATES,
Acting Commissioner of Internal Revenue.

(From California State Journal of Medicine.)

---

**Scientific Glass,** for microscopic lenses, has been produced by the Spencer Lens Co. of Buffalo, for the first time in America. This is one phase of the issue of industrial American independence, stimulated by the war and alluded to editorially last year. Glass of sufficient quality for spectacles, was made in America for the first time six months ago.

---

**A National Campaign to Reduce Infant Mortality** was instituted by the American Association for the Study and Prevention of Infant Mortality, at Philadelphia, November 12. The week March 4-11, has been set for popular instruction, and national legislation regarding child labor is advocated. It should be noted that while certain philanthropic campaigns require elaborate means of acquiring knowledge, one of this nature depends mainly on the inculcation and practical execution of principles already well known professionally and even by the intelligent laity.

**Fraudulent Neosalvarsan.** Several shipments of worthless imitation drug products have been seized by the officials of the U. S. Dept. of Agriculture, including a preparation labeled Neosalvarsan, which proved to consist of a solution of sodium chlorid colored with a coal tar dye.

**Sterilization** has been enforced in Wisconsin, 24 patients in the Institution for the Feeble Minded at Chippewa Falls, having been castrated.

**A County Hospital for Tuberculous Patients** was voted by Nigara County, N. Y., by a plurality of nearly 8,000. Jefferson, Rockland, Steuben and Herkimer Counties also voted for similar institutions.

**The Tonawanda City Hospital** for the year ended September 30, showed a saving of over $1,000 as compared with gross receipts. For the fiscal year ended September 30, 1914, the saving was $275. The per capita cost has been reduced from 68 to 50 cents for food and from $2.43 to $2.18 total.

**Arnold Gregory Memorial Hospital of Albion.** The corner stone was laid, without ceremony, November 12.

**New Vehicle Ordinance for Buffalo.** All vehicles passing schools and churches at dismissal time must reduce speed to for similar institutions. Niagara Co. will spend about $100,-000 for this purpose.

**College Merger.** Steps have been taken to merge the Medico-Chirurgical Medical College of Philadelphia, with the Medical Department of the University of Pennsylvania.

**Elmira** is having a mild typhoid epidemic, fifteen cases having developed on one milk dealer's route.

**Bequests to Medical Charities.** By the will of Mrs. Josephine L. Goodyear of Buffalo, $20,000 is left for a Convalescent Home, named for the donor, and $5,000 each to the Buffalo Homoeopathic Hospital and the Children's Hospital. Most commendable is the bequest of sums of $2,000 to $10,000 to domestic employees.

**Deliberately Allowing a Defective Child to Die,** is charged against and proudly admitted by Dr. H. J. Haiselden of Chicago. Much publicity has been given the case and a stranger, learning of the facts, attempted to kidnap the child to have treatment instituted with the hope of saving the life. The parents consented to the abandonment of the child to its fate. Lack of one ear, deformity of the other, absent or undeveloped cranial nerves, "absence of neck," and deformity of chest and of nose, are stated to be the reasons for allowing the child to die. It is also stated that the brain was slightly subnormal, though press despatches do not say whether this diagnosis was made ante or post mortem. Occlusion of the bowel is also hinted at. It is obvious that the viability and mental and physical potentialities of the child, aside from purely aesthetic drawbacks, cannot be determined except by careful and very direct examination. It is pretty well recognized that an absolutely hopeless case, whether of the new born or at any time during life, may be allowed to die, provided that operative or other measures of relief produce discomfort, or involve immediate increase of danger of death, and do not hold out hopes of more than very transient prolongation of life. It is equally well recognized that the mere undesirability of life, whether from the standpoint of the sociologist, physician, family or the patient himself, so far as it can be determined, does not justify neglect of all means for preserving and prolonging life.

The coroner's jury, consisting of Drs. John F. Golden, Arthur Rankin, D. Howland Chislett, D. A. K. Stele, Henry F. Lewis and Ludwig Hektoen, held that the doctor was justified in allowing the baby to die. Dr. Haiselden stated that he had consulted 15 physicians, 14 of whom concurred in his opinion. Dr. Dill Roberts, City Health Commissioner, testified that there was no evidence that the child would be mentally defective, and there seemed to be doubt of total blindness and deafness. Dr. Roberts also held that several of the physical defects could have been remedied by plastic operations. The acquittal, therefore, is notable not because it was based on the non-viability of the infant, but as upholding the moral and ethical right of a physician and surgeon to refuse to perform an operation not sanctioned by his conscience. The verdict explicitly recommends consultation in similar cases, but finds that the right of consultation was not denied the parents. Indeed, it appears that the recommendation was more than complied with. It will be interesting to note whether this verdict will be used as a precedent in future cases, and whether it will lead to an extension of application in the direction of euthanasia in hopeless cases.

---

**Child Suicide.** A case in a boy of 15, is reported from near

Corning. The boy was compelled to suport himself by working on a farm, and he had been notified that he would be discharged. He bought, or somehow secured, a revolver, asked his mistress for a cartridge with which to shoot himself, and she gave him one, saying that it would be a good thing—in joke of course. How any sane adult could imagine that a boy of 15, at any rate without careful supervision, should be allowed to have a revolver in his possession is one question that presents itself. How it could be assumed that suicide would be regarded as a joke by a homeless child, presumably already depressed by the premature assumption of the responsibilities of self-support and with lack of employment at this season staring him in the face, is another question that demands an answer. Sentiment aside, if the boy was mentally, morally or physically defective, there is an abundance of places provided for his care. If he was anywhere near normal in these respects, he was worth his support and schooling, to almost any farmer and to many other persons, and it should have been an easy matter to have assured him on this point and to have found him a place. Here is a good opportunity to impress the fact that plain kindness of heart has a strong influence on such technical matters as mortality rates, disease incidence and industrial self-sufficiency.

---

**Subspecialism.** Among 30 physicians carrying professional cards in the Texas Medical Journal, 2 announce that their practice is limited to PELLAGRA.

---

**The Sherley Amendment to the U. S. Food and Drugs Act** has been held constitutional by the U. S. Court for the eastern district of Pennsylvania. This amendment forbids placing on the label false and fraudulent statements regarding curative or therapeutic effects. It occurs to us that this ruling requires further qualification. There are many drugs concerning which there is marked difference of opinion, for example cactus. A very small minority of the medical profession holds, in perfect faith, quite a number of drugs which are usually regarded as impotent. Again, some very simple remedy, not necessarily a drug, may indirectly produce a very favorable action tending to support the heart or to affect some other vital apparatus. Occasionally, apparently incontrovertible pharmacologic evidence upsets notions long and generally held. For instance, suppose a physician dispenses a package of strychnine tablets, marked with the name of the drug, the amount in each tablet, the usual directions as to taking, and adds "Heart Tonic;" or suppose he similarly marks a package of calomel tablets "To promote flow of bile." What would be his legal status, in case some enemy or some newly conceived not to depart from the field of commercialism, will this amendment not be susceptible of abuse in regard to firms issuing, to supply an actual professional demand, certain drugs that are not in scientific favor but which practitioners, in greater or less number, consider efficient in certain conditions?

